# Supplementary material for: Analysis of Cytosine Base Editors in Bovine Zygotes: Efficiency and Editing Window Characterization Through Targeting the MYO7A Gene
Source: Curr Issues Mol Biol. 2025 Dec 11;47(12):1033. doi: 10.3390/cimb47121033 (PMC12732011; doi:10.3390/cimb47121033)
Supplement: Supplementary file 1 [file cimb-47-01033-s001.zip › cimb-3948303-supplementary.pdf]

				20	19	18	17	16	15	14	13	12	11	10	9	8	7	6	5	4	3	2	1										
	PAM			sgRNA																												Mutation	
WT	C	C	C	A	C	T	T	C	A	G	T	C	C	A	C	G	G	C	G	T	G	G	A	G	G	A	C	A	T	G	A		
Blastocyst 1																a	Y		Y		Y	a		a	a					a		Y	
Blastocyst 2																Y	Y		Y		Y	a										Y	
Blastocyst 3																Y	Y		Y		Y	Y		a	a					Y		All	
Blastocyst 4																Y	Y		a		Y	Y										Y	
Blastocyst 5																Y	Y		Y		Y	Y		a	a					a		All	G-to-A
Blastocyst 6																Y	Y		a		a	N										Y	
Blastocyst 7																Y	Y		Y		Y	Y		Y	Y							All	
Blastocyst 8																Y	Y		Y		Y	Y		a	a					a		All	Indel
Blastocyst 9																Y	Y		Y		a	a			a					a		Y	
Blastocyst 10																Y	Y		Y		a	a										Y	
Blastocyst 11																Y	Y		Y		a	a										Y	
Arrested Embryo 1																Y	Y		a		Y	Y										Y	
Arrested Embryo 2																a	a		a		a	a										Y	
Arrested Embryo 3																a	a		a		a	a										Y	
Complete conversion efficiency																0.79	0.86		0.64		0.57	0.43											
Incomplete conversion efficiency																0.21	0.14		0.36		0.43	0.50											
No conversion efficiency																0.00	0.00		0.00		0.00	0.08											
Compltet conversion efficiency in blastocyst																0.91	1.00		0.82		0.64	0.45											
Compltet conversion efficiency in arrested embryo																0.33	0.33		0.00		0.33	0.33											

Supplementary Figure S1: C-to-T base editing outcomes in bovine embryos from the BE3 group. Sanger sequencing results for individual blastocysts and arrested embryos from BE3 group. The top sequence indicated the wild-type reference sequence for the targeted genomic region. The PAM sequence (NGG) is highlighted in red. The 20-nucleotide sgRNA target sequence is highlighted in yellow, with nucleotide positions numbered 1–20 from the PAM-distal end. The five target cytosines within this sequence are located at positions 2, 3, 5, 7, and 8. The editing outcome at each of the five target cytosine positions is categorized as Y (green) is CC, a (gray) is IC, and N (yellow) is NC. The "Mutation" column indicated the detection of conversion. Y indicates at least one C-to-T conversion detected. All represents all five target cytosines resulted in CC. Indel indicates that an indel mutation was identified at the target site. G-to-A showed complementary DNA strand conversion detection.

				20	19	18	17	16	15	14	13	12	11	10	9	8	7	6	5	4	3	2	1										
	PAM			sgRNA																												Mutation	
WT	C	C	C	A	C	T	T	C	A	G	T	C	C	A	C	G	G	C	G	T	G	G	A	G	G	A	C	A	T	G	A		
Blastocyst 1																Y	Y		Y		a	a		a	a							Y	
Blastocyst 2																Y	Y		a		a	N			a							Y	
Blastocyst 3																Y	Y		Y		N	N										Y	
Blastocyst 4																Y	a		a		a	a										Y	
Blastocyst 5																Y	a		a		Y	N		a	a							Y	
Blastocyst 6																a	a		a		a	N										Y	
Blastocyst 7																Y	Y		Y		Y	Y										All	
Blastocyst 8																Y	a		a		a	a										Y	
Blastocyst 9																a	a		Y		a	a		a	a							Y	
Blastocyst 10																Y	Y		Y		a	N										Y	
Blastocyst 11																a	Y		Y		a	a										Y	
Blastocyst 12																a	a		Y		a	a										Y	
Blastocyst 13																Y	a		Y		Y	a										Y	
Blastocyst 14																a	a		a		a	a		a	a							Y	Indel detection
Blastocyst 15																Y	a		Y		a	a										Y	
Blastocyst 16																Y	a		Y		Y	Y										Y	
Arrested Embryo 1																a	a		a		a	N										Y	
Arrested Embryo 2																a	a		Y		Y	Y										Y	
Arrested Embryo 3																Y	Y		Y		N	N										Y	
Complete conversion efficiency																0.63	0.37		0.63		0.26	0.16											
Incomplete conversion efficiency																0.37	0.63		0.37		0.63	0.47											
No conversion efficiency																0.00	0.00		0.00		0.11	0.39											
Compltet conversion efficiency in blastocyst																0.69	0.38		0.63		0.25	0.13											
Compltet conversion efficiency in arrested embryo																0.33	0.33		0.67		0.33	0.33											

Supplementary Figure S2: C-to-T base editing outcomes in bovine embryos from the BE3-Y130F group. Sanger sequencing results for individual blastocysts and arrested embryos from BE3-Y130F group. The top sequence indicated the wild-type reference sequence for the targeted genomic region. The PAM sequence (NGG) is highlighted in red. The 20-nucleotide sgRNA target sequence is

highlighted in yellow, with nucleotide positions numbered 1–20 from the PAM-distal end. The five target cytosines within this sequence are located at positions 2, 3, 5, 7, and 8. The editing outcome at each of the five target cytosine positions is categorized as Y (green) is CC, a (gray) is IC, and N (yellow) is NC. The "Mutation" column indicated the detection of conversion. Y indicates at least one C-to-T conversion detected. All represents all five target cytosines resulted in CC. Indel indicates that an indel mutation was identified at the target site.

Supplementary Table S1. Primer information for Cas9, BE3, and BE3-Y130F mRNA production.

Primer name	Sequence (5'-3')	
Cas9-F	TAATACGACTCACTATAGGGAGAATGGACTATAAGGACC ACGAC	
Cas9-R	GCGAGCTCTAGGAATTCTTAC	
Base-Editor-F	TAATACGACTCACTATAGGGAGAGCCGCCACCATG	Used for BE3 and BE3-Y130F
Base-Editor-R	CAGCGGGTTTAAACTCAATGGTGATG	
